# Multimode MALDI-MSI deciphers matrine-induced metabolic reprogramming in prostate cancer xenografts: spatial mapping of low-molecular-weight compound alterations

**DOI:** 10.3389/fphar.2025.1627864

**Published:** 2025-12-18

**Authors:** Jia Xu, Liang Qin, Xiao Liang, Lulu Chen, Juexin Wang, Hua Guo, Fengmei Wang, Ran Wu, Xiaojing An, Wenjuan Liu, Xiaodong Wang, Qi Li

**Affiliations:** 1 Department of Clinical Laboratory, Xiyuan Hospital, China Academy of Chinese Medical Sciences, Beijing, China; 2 Centre for Imaging & Systems Biology, Minzu University of China, Beijing, China; 3 College of Life and Environmental Sciences, Minzu University of China, Beijing, China; 4 Department of Pathology, Xiyuan Hospital, China Academy of Chinese Medical Sciences, Beijing, China; 5 College of Biological Sciences and Technology, Beijing Forestry University, Beijing, China

**Keywords:** prostate cancer, matrine, matrix-assisted laser desorption/ionization mass spectrometry imaging, low-molecular-weight compound, metabolic reprogramming

## Abstract

**Background:**

Matrine, a bioactive isoquinoline alkaloid, exhibits antitumor efficacy by modulating multiple signaling pathways to suppress cancer cell proliferation, migration and invasion. However, its metabolic regulatory mechanisms in prostate cancer intervention require systematic characterization.

**Methods:**

We implemented matrix-assisted laser desorption/ionization mass spectrometry imaging (MALDI-MSI) for spatial metabolomic profiling of prostate tissues, integrated with multivariate analytical approaches including principal component analysis, Pearson correlation-based clustering heatmap, partial least squares-discriminant analysis, and hierarchical clustering heatmap analysis. This multimodal strategy enabled comparative evaluation of low-molecular-weight metabolite distributions across normal control, prostate cancer, and matrine-treated prostate cancer cohorts.

**Results:**

Multi-omics integration identified 19 discriminant metabolites (VIP >1.0) spanning lipid signaling mediators (choline, glycerophosphoglycerol, sphinganine, glycerophosphoinositol, linoleic acid, oleic acid, N,N-Dimethylsphingosine), amino acid network regulators (cysteic acid, 5-Hydroxylysine, glutamine-glutamate axis components), nucleotide biosynthesis (adenine, Ribose 1,5-bisphosphate, uracil, dihydrouracil, deoxyinosine, adenosine), markers of oxidative damage (8-Hydroxyguanine) and cofactor of nitric oxide synthases and aromatic amino acid hydroxylases (tetrahydropteridine). Linoleic acid, oleic acid, and N,N-Dimethylsphingosine exhibited the highest levels in the NC group; these metabolites were significantly downregulated in the PCa group and partially restored in the PCa+MAT group. In addition, the results revealed a progressive depletion of tetrahydropteridine across experimental groups, with the PCa+MAT group exhibiting significantly lower tetrahydropteridine levels compared to both NC and PCa groups (PCa+MAT < NC < PCa). Notably, the expression levels of other compounds were the lowest in the NC group, while they were significantly upregulated in the PCa group and with intermediate levels observed in the PCa+MAT group. Spatial metabolomics delineated dynamic metabolic reprogramming during prostate cancer progression, with matrine treatment demonstrating partial reversal of cancer-associated metabolic shifts, particularly in lipid pathways, underscoring its potential as a modulator of oncogenic metabolism.

**Conclusion:**

This study establishes MALDI-MSI as a powerful platform for pharmacometabolomic evaluation, while elucidating matrine’s therapeutic potential through coordinated regulation of lipid metabolic remodeling, amino acid/nucleotide biosynthesis pathways and oxidative stress responses. Our findings provide mechanistic insights into matrine’s anticancer action and validate metabolomic approaches for natural product evaluation.

## Graphical Abstract

**Figure:**
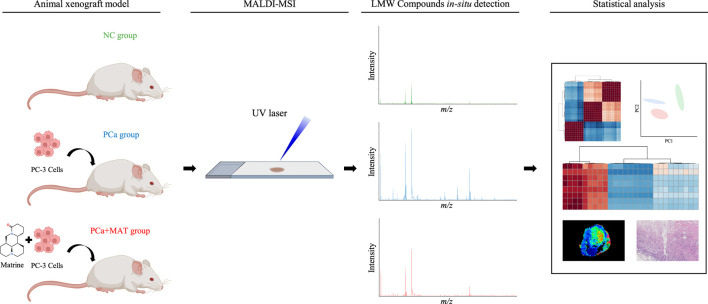

## 1 Introduction

Prostate cancer (PCa) is the most common malignant tumor of the male reproductive system and the second most common cancer in the world, which poses a serious threat to the survival of middle-aged and elderly men worldwide (Bergengren et al., 2023; Bray et al., 2024). The epidemiological pattern of this cancer demonstrates a significant age-associated incidence gradient, with clinical outcomes critically contingent upon the therapeutic window encompassing diagnostic confirmation and intervention (Siegel et al., 2024). Although the incidence of PCa in China is lower than that in the west, it is also showing an increasing trend year by year with the aging population, environmental changes and the improvement of diagnostic technology (Li et al., 2019). Recent advances in PCa research have significantly expanded our understanding of disease progression and therapeutic resistance, particularly in castration-resistant prostate cancer (CRPC), including the growing recognition of the therapeutic potential of natural compounds that target oncogenic signaling pathways to overcome drug resistance. For instance, caffeic acid has been shown to suppress proliferation and induce apoptosis by inhibiting the Interleukin-6 (IL-6)/Janus kinase (JAK)/Signal transducer and activator of transcription-3 (STAT-3) signaling axis (Yin et al., 2024), while the flavonoid chrysin acts as an antiandrogen with specific cytotoxicity toward PCa cells harboring mutant androgen receptors (e.g., AR-T877A and AR-W741L), which are often linked to resistance against conventional antiandrogens in CRPC (Carranza-Aranda et al., 2024). Beyond cancer cell-autonomous mechanisms, the tumor microenvironment (TME) has emerged as a critical regulator of disease progression and therapy resistance. Studies using extracellular matrix (ECM)-mimicking hydrogel models reveal that adipocytes from various fat depots engage in metabolic crosstalk with PCa cells under antiandrogen treatment, leading to deregulated lipid metabolism and adipokine secretion that foster a resistant niche (Bessot et al., 2025). Additionally, novel oncogenic drivers such as Transmembrane emp24 trafficking protein 3 (TMED3) have been identified, which promote tumor progression by inactivating tumor-suppressive forkhead box O transcription factor (FOXO) transcription factors (Wei et al., 2025). In parallel, innovative therapeutic strategies like proteolysis-targeting chimeras (PROTACs) are being developed to degrade androgen receptor variants and overcome resistance mechanisms (Zhang et al., 2024). Furthermore, the influence of aging and systemic inflammation on PCa aggressiveness is increasingly recognized, with aging-associated secretory phenotypes and pro-inflammatory cytokines such as IL-6, interleukin-8 (IL-8), and C-X-C motif (CXC) chemokines contributing to immune evasion and metastasis (Ullah et al., 2024). These multifaceted insights underscore the complexity of PCa progression and highlight the need for integrative approaches targeting metabolic reprogramming, signaling pathways, and the TME.

At present, it is believed that traditional Chinese medicine (TCM), as an alternative therapy for cancer, can improve the quality of life and survival time of cancer patients. Botanical-derived compounds are gaining recognition as promising adjunctive therapeutics in oncology due to their multi-target mechanisms and reduced off-target toxicity compared to conventional chemotherapeutics. Among these natural agents, matrine—a tetracyclic quinazoline alkaloid isolated from *Sophora* species—has emerged as a prototypical candidate bridging traditional medicinal knowledge and modern molecular oncology. Matrine is the main bioactive component of *Sophora flavescens* Alt. and *Sophora tonkinensis* roots, it can easily penetrate the biofilm barrier and produces a wide range of pharmacological effects, including anti-inflammatory, antioxidant, anti-cancer, neuroprotective, alter gut microbiota composition, etc. (Zhang et al., 2020). Studies have demonstrated that matrine inhibits cancer cell proliferation, invasion, and metastasis, and induces apoptosis and autophagy across various cancer types, including hepatocellular carcinoma, gastric cancer, breast cancer, and leukemia (Lin et al., 2022). Its anti-tumor mechanisms involve the regulation of multiple signaling pathways, such as phosphatidylinositol 3-kinase/protein kinase B/mammalian target of rapamycin (PI3K/AKT/mTOR), nuclear factor kappa B (NF-κB), Wnt (wingless/integration 1)/β-catenin, mitogen-activated protein kinases (MAPKs) (Lin et al., 2022). Furthermore, matrine can reverse multidrug resistance by downregulating P-glycoprotein and MRP1 expression (You et al., 2020). However, the clinical application of matrine is constrained by its notable limitations. Serious adverse effects, including hepatotoxicity and neurotoxicity, have been reported, which are major factors limiting its clinical use (Li et al., 2021). Pharmacokinetic studies reveal that matrine suffers from low oral bioavailability and a short half-life *in vivo* (Sun et al., 2022). Different administration routes significantly affect its absorption and distribution, with intramuscular injection providing better bioavailability than oral administration (You et al., 2020). Many pharmacological effects of matrine, especially its anti-cancer properties, are not isolated but can be synergistic when combined with other drugs. For instance, matrine enhances the efficacy of chemotherapeutic agents like cisplatin (Hu et al., 2021), 5-fluorouracil (Hu et al., 2022), and docetaxel (Li et al., 2019), showing synergistic inhibition of cancer cell proliferation and induction of apoptosis. These synergistic interactions highlight matrine’s potential as an adjunctive therapy in combination regimens. Our prior mechanistic investigation employing high-throughput sequencing revealed matrine’s profound regulatory effects on prostate cancer pathophysiology. Functional enrichment analysis of differentially expressed genes in PC-3 and DU145 cell lines identified significant perturbations in core oncogenic processes, including cell cycle regulation, apoptotic signaling, and metabolic pathway modulation. Kyoto Encyclopedia of Genes and Genomes (KEGG) pathway mapping further demonstrated matrine’s preferential targeting of the PI3K-AKT signaling pathway, with concomitant downregulation of PI3K (Li et al., 2018). These findings positioned matrine as a potent modulator of androgen-independent prostate cancer progression through coordinated inhibition of proliferative signaling and metabolic reprogramming. Despite these extensive pharmacological findings, significant knowledge gaps remain regarding the precise molecular mechanisms underlying matrine’s anti-cancer effects, particularly in the context of PCa. Furthermore, the detailed metabolic consequences and the specific alterations in low-molecular-weight (LMW) compounds induced by matrine treatment in PCa tissues are poorly understood. Elucidating these aspects is crucial for fully harnessing matrine’s therapeutic potential and optimizing its clinical application.

Multimodal Matrix-assisted laser desorption/ionization mass spectrometric imaging (MALDI-MSI) is a label-free molecular profiling technique that permits the direct determination of the spatial distribution and relative abundance of a wide range of molecules, including endogenous and exogenous compounds, peptides, and proteins in a biological sample such as tissue specimen (Chen et al., 2023). Low-molecular-weight (LMW) compounds perform multiple essential functions in multitudinous biological processes, including energy transformation, signal transduction and regulation, etc. The changes of LMW compounds in tumors reflect multiple aspects such as metabolic reprogramming of tumor cells, genetic mutations, changes in tumor microenvironment (Zhu, 2024), and therapeutic response (Navarro et al., 2023). These changes not only help us understand the occurrence and development of tumors, but also can provide targets for new therapeutic strategies. In-depth study of the changes in these LMW compounds can provide valuable clues for the development of more precise therapeutic strategies and new drugs.

Building upon these insights, the present study employed MALDI-MSI coupled with multivariate statistical analysis to systematically investigate matrine-induced alterations of LMW compounds in prostate cancer xenografts. The investigation was structured around three primary objectives: (1) to determine the feasibility of MALDI-MSI-based LMW compound profiling in discriminating between normal control (NC), PCa, and matrine-treated PCa (PCa+MAT) groups, (2) to analyze the structural identification and expression characteristics of differential LMW compounds; and (3) to elucidate the molecular mechanism of matrine affecting the metabolism of LMW compounds in prostate cancer by comprehensive metabolomics analysis. The findings derived from this investigation will advance our mechanistic understanding of matrine-mediated regulatory pathways in prostate carcinogenesis, specifically elucidating its capacity to modulate LMW metabolite networks in PCa tissues. Concurrently, this work establishes MALDI-MSI-based metabolomics as an emerging analytical paradigm for rapid, reliable assessment of matrine-induced therapeutic metabolic reprogramming in pharmacological interventions against PCa.

## 2 Materials and methods

### 2.1 Reagents and materials

Three compounds of matrine, 2-mercaptobenzothiazole (2-MBT), and Michler’s ethylketone (MEK) were purchased from Sigma-Aldrich (St. Louis, MO). LC-MS grade methanol (MeOH), trifluoroacetic acid (TFA), ammonia hydroxide (NH_3_∙H_2_O), and hematoxylin and eosin (H&E) stain solutions were also purchased from Sigma-Aldrich (St. Louis, MO). Ultrapure water used throughout the experiments was obtained from the Milli-Q system (Millipore, United States). Six-week-old male BALB/c nude mice were purchased from Charles River Laboratories (Beijing, China). Human prostate cancer cell lines PC-3 were purchased from the Experimental Animal Center of Sun Yat-sen University.

### 2.2 Cell culture

Human prostate cancer cell lines PC-3 were cultured with RPMI-1640 medium supplemented with 10% fetal bovine serum (Invitrogen, United States), 100 units/mL penicillin (Life Technologies, United States) and 100 μg/mL streptomycin (Life Technologies, United States). Cells were cultured in the incubator (5% CO_2_, 37 °C).

### 2.3 Animal xenograft model assays

Male BALB/c nude mice (6-weeks old) were raised under specific pathogen-free (SPF) conditions. Mice were anesthetized with isoflurane and placed in a supine position and the skin is disinfected using iodophor. A longitudinal incision approximately 1 cm in length was made in the middle of the lower abdomen. The preputial glands are carefully separated, followed by incising the abdominal muscle layer to expose the bladder and the left seminal vesicles. For the PCa and PCa+MAT groups, suspensions of PC-3 cells (0.1 mL 5 × 10^7^ viable cells/mL) were slowly injected along the direction of the bladder. For the NC group, an equal volume of sterile saline (0.1 mL) was injected following the same procedure to serve as a sham operation control. During the injection process, a pause was made, and then the needle was carefully removed. After confirming that there was no leakage of the cell suspension, the muscle layer and the skin were closed and sutured. After that, the gas anesthesia machine was turned off, and the mice were placed on a heating pad to facilitate recovery. Once the animal regains consciousness, it was transferred back to the cage. Nine days later, the tumor volumesin the PCa and PCa+MAT groups reached ∼100 mm^3^. The NC group did not develop tumors, confirming the absence of tumorigenic injection. The PCa+MAT group was subjected to intraperitoneal injection of 200 mg/kg matrine, while the PCa and NC groups received intraperitoneal injection of an equal volume of saline three times per week (6 mice in each group). Tumor sizes were measured twice weekly, and the volumes (cm^3^) were calculated according to the formula: v = (length × width^2^)/2. Animals of three groups were sacrificed and the prostate tissue was dissected 3 weeks later. All tissue samples were slowly immersed in liquid nitrogen to avoid shattering after harvest and then stored at −80 °C until used. All animal experiments were carried out according to the Guidelines for the Care and Use of Laboratory Animals and were performed under the supervision of the Ethics Committee of Xiyuan Hospital of China Academy of Chinese Medical Sciences and the committee on biological and medical ethics of Minzu University of China (ECMUC2020004AA).

### 2.4 Tissue sectioning

The frozen prostate tissues were cryo-sectioned at −20 °C into 20 μm thick slices in a Leica CM1860 cryostat (Leica Microsystems Inc.). The serial tissue slices were then immediately thaw-mounted on the conductive sides of indium-tin oxide (ITO)-coated microscope glass slides (Bruker Daltonics, Bremen, Germany).

### 2.5 Matrix coating

2-MBT was prepared at a concentration of 7 mg/mL in a mixed 80:20 (v/v) MeOH: water containing 0.2% TFA. MEK was prepared at a concentration of 12 mg/mL dissolved in 90% MeOH containing 1.0% NH_3_∙H_2_O. Using a HIT MatrixPrep (Beijing Huayi Innovation and Biotechnology Co., Ltd., Beijing, China) sprayed the matrix solution onto the tissue sections. The matrix solution was sprayed on the tissue sections for two cycles. For matrix coating, the matrixes solution was applied to the surfaces of the sliced tissue sections using a HIT MatrixPrep at a density of 0.18 mg/cm^2^ over a period of 8 min. The flow rate of the matrix solution was 200 μL per minute, and the gas pressure was maintained at 0.65 MPa.

### 2.6 MALDI-MSI

All of the profiling and imaging experiments were performed using a Bruker Autoflex Speed MALDI time-of-fight (TOF)/TOF mass spectrometer (Bruker Daltonics). The MALDI source was equipped with a 2,000 Hz solid-state Smartbeam Nd:YAG UV laser (355 nm, Azura Laser AG). All the mass spectra were acquired over a mass range of *m*/*z* 100–500 in both positive and negative ion modes with broadband detection. For the acquisition of MALDI-MSI profiling data, the mass spectra were recorded from an accumulation of 20 laser scans, and each scan was accumulated from 500 laser shots. For imaging data acquisition, 75 μm laser raster step-sizes were used for the *in situ* detection of endogenous LMW compounds in the prostate tissue sections, and each scan (pixel) was accumulated from 500 laser shots. On the basis of the use of Bruker’s FlexImaging 4.1 software, a correction pen was used to mark the “teaching points” (generally three points) around a tissue section for the correct positioning of the UV laser for the spectral acquisition. In the positive ion mode, the ions of Pro ([M+H]^+^, *m*/*z* 116.07), 3,4 dimethoxycinnamic acid (DMCA) ([M+H]^+^, *m*/*z* 209.08), 2-MBT ([M+H]^+^, *m*/*z* 167.99), α-cyano-4-hydroxycinnamic acid (CHCA) ([M+H]^+^, *m*/*z* 190.05), CHCA ([M+Na]^+^, *m*/*z* 212.03), CHCA ([2M+H]^+^, *m*/*z* 379.09), CHCA ([3M+H]^+^, *m*/*z* 568.13), were used for external mass calibration. In the negative ion mode, a mixed matrix and standard solution, including 2,5-dihydroxybenzoicacid (DHB) ([M-H]^-^, *m*/*z* 153.02), CHCA ([M-H]^-^, *m*/*z* 188.04), sinapinic acid (SA) ([M-H]^-^, *m*/*z* 223.06), MEK([M-H]^-^, *m*/*z* 323.21), SA ([2M-H]^-^, *m*/*z* 447.13), were used for external mass calibration. The cubic enhanced mode was chosen for both external and internal mass calibration processing.

### 2.7 MS/MS validation and data analysis

To confirm the identities of the discriminant LMW compounds, on-tissue tandem MS (MS/MS) experiments were performed directly on the tissue sections using the MALDI-TOF/TOF instrument. The acquired MS/MS spectra were processed using FlexAnalysis 3.4 software. The key precursor ions listed in Table 1 were selectively fragmented using collision-induced dissociation (CID). The resulting MS/MS spectra were then matched against the MS/MS spectral libraries within the HMDB database. This fragmentation data provided orthogonal confirmation for the initial assignments based on accurate mass. Three ion forms ([M+H]^+^, [M+Na]^+^ and [M+K]^+^) for the positive ion mode and two ion forms ([M-H]^−^ and [M+Cl]^−^) for the negative ion mode were allowed during the database searching. Reconstruction of the ion maps of detected LMW compounds was performed by using the Bruker FlexImaging 4.1 software.

**TABLE 1:** List of significantly differential LMW compounds (VIP>1) detected and identified in the prostate tissues from NC, PCa and PCa+MAT groups, by MALDI-TOF MS in the positive and negative ion modes.

Measured *m*/*z*	Calculated *m*/*z*	Error (ppm)	Assignment			Structurally specific CID ions (*m*/*z*)
			Ion form	Compound	Molecular formula	MALDI-MS/MS
104.107	104.1075	5	[M+H]^+^	Choline	C5H14NO	104, 60
136.062	136.0618	2	[M+H]^+^	Adenine	C5H5N5	135, 118, 92
170.012	170.0118	1	[M+H]^+^	Cysteic acid	C3H7NO5S	124, 105
185.089	185.0897	4	[M+Na]^+^	5-Hydroxylysine	C6H14N2O3	128, 82
269.042	269.0397	9	[M+Na]^+^	Glycerophosphoglycerol	C6H15O8P	247, 229, 173
302.305	302.3054	1	[M+H]^+^	Sphinganine	C18H39NO2	302, 284
332.975	332.9747	1	[M+Na]^+^	Ribose 1,5-bisphosphate	C5H12O11P2	310, 213, 115
335.074	335.0738	1	[M+H]^+^	Glycerophosphoinositol	C9H19O11P	173, 155
111.020	111.0200	0	[M-H]^−^	Uracil	C4H4N2O2	111
113.035	113.0357	6	[M-H]^−^	Dihydrouracil	C4H6N2O2	112, 68
135.067	135.0676	5	[M-H]^−^	Tetrahydropteridine	C6H8N4	108, 94
145.062	145.0619	1	[M-H]^−^	Glutamine	C5H10N2O3	127, 109, 84
146.046	146.0459	1	[M-H]^−^	Glutamate	C5H9NO4	128, 102
166.037	166.0370	0	[M-H]^−^	8-Hydroxyguanine	C5H5N5O2	138, 123, 41
251.079	251.0786	2	[M-H]^−^	Deoxyinosine	C10H12N4O4	135, 108
266.090	266.0895	2	[M-H]^−^	Adenosine	C10H13N5O4	134, 107
279.233	279.2330	0	[M-H]^−^	Linoleic acid	C18H32O2	279
281.249	281.2486	1	[M-H]^−^	Oleic acid	C18H34O2	281
326.307	326.3065	2	[M-H]^−^	N,N-dimethylsphingosine	C20H41NO2	296, 237

VIP, variable importance of projection.

### 2.8 Histological staining

After MALDI-MSI, the tissue sections were washed with different concentrations of EtOH. To obtain histological images of prostate tissue sections, H&E staining was performed according to a previously reported procedure (Casadonte and Caprioli, 2011). Based on H&E staining, cancerous and non-cancerous regions can be easily distinguished by their different staining colors.

### 2.9 Microscopy visualization

An Epson Perfection V550 Photo Scanner (Epson Inc., Suwa, Japan) was used to obtain optical images of the tissue sections.

### 2.10 Statistical analysis

The monoisotopic peak lists obtained from processing individual mass spectra were saved as individual CSV text files. The MetaboAnalyst 6.0 platform (https://www.metaboanalyst.ca/) was used for the principal component analysis (PCA), Pearson correlation-based clustering heatmap analysis, Partial least squares-discriminant analysis (PLS-DA), hierarchical clustering heatmap analysis, and to evaluate the expression changes of LMW compounds in prostate tissues from NC, PCa and PCa+MAT groups detected by MALDI-MS. GraphPad Prism version 8.0 was employed for generating violin plots to visualize data distribution. Statistical analysis was performed by one-way analysis of variance (ANOVA) using SPSS version 25 (IBM Corp., Armonk, United States). “*”, 0.01 < *p* < 0.05; “**”, 0.001 < *p* < 0.01; “***”, *p* < 0.001.

## 3 Results

### 3.1 Comparison of LMW compounds in-situ detection in prostate tissues from NC, PCa and PCa+MAT groups by MALDI-TOF MS in positive and negative ion modes

**FIGURE 1:**
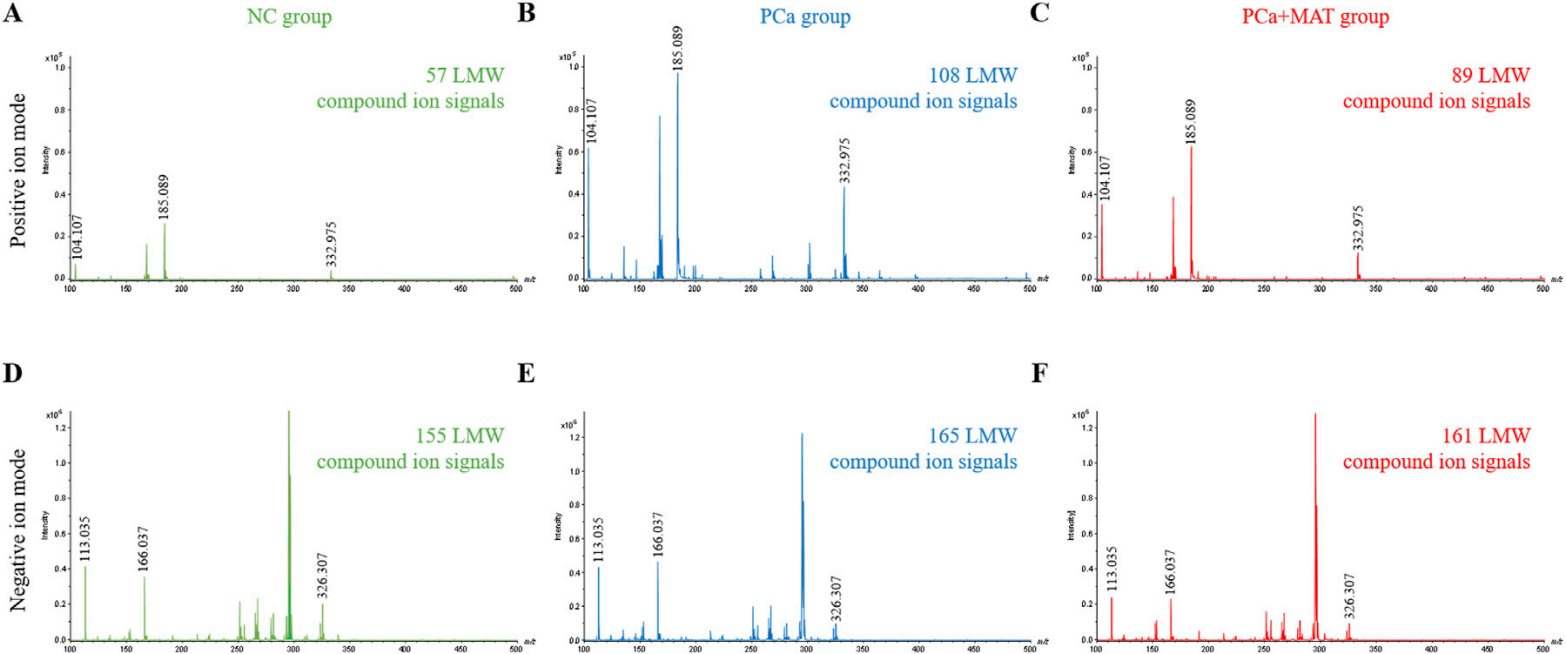
Comparison of mass spectra of *in situ* detectable LMW compounds in prostate tissues from NC **(A,D)**, PCa **(B,E)** and PCa+MAT groups **(C,F)**, by MALDI-TOF MS in the positive and negative ion modes using 2-MBT and MEK as the matrices.

Prostate tissues from NC, PCa, and PCa+MAT groups were adhered to conductive ITO glass slides for *in situ* detection of LMW compounds using MALDI-TOF MS in both positive and negative ion modes with 2-MBT and MEK matrices (Figure 1). A range of LMW compound-related ion signals (*m*/*z* 100–500) were clearly detected across all groups. In positive ion mode (Figures 1A–C), 57, 108, and 89 unique LMW compound ion signals were detected in NC, PCa, and PCa+MAT tissues, respectively. Notably, significant intergroup differences were observed not only in the quantity of detectable LMW compounds but also in the expression abundance of specific ions (e.g., *m*/*z* 104.107, *m*/*z* 185.089, *m*/*z* 332.975). In contrast, negative ion mode analysis (Figures 1D–F) revealed comparable total ion counts (155 in NC group, 165 in PCa group, 161 in PCa+MAT group), yet displayed marked variations in expression levels of key metabolites (e.g., *m*/*z* 113.035, *m*/*z* 166.037, *m*/*z* 326.307).

These findings demonstrate distinct mass spectral profiles between groups, characterized by differential LMW compound quantities, peak patterns, and expression intensities. The metabolic signature of PCa+MAT group exhibited greater similarity to NC than PCa group, suggesting partial restoration of LMW compound homeostasis following matrine intervention. The above results collectively confirm that matrine significantly modulates LMW compound metabolism in prostate carcinogenesis, particularly through partial restoration of metabolic patterns toward physiological baselines. While this study establishes MALDI-TOF MS as an effective platform for metabolomic profiling of matrine’s therapeutic effects, further investigations are warranted to elucidate the precise molecular mechanisms underlying these metabolic reprogramming events.

### 3.2 Multivariate analysis of LMW compound profiles through PCA and pearson correlation-based clustering heatmap

To statistically evaluate metabolic differences between groups, PCA and Pearson correlation-based clustering heatmap were applied to MALDI-MS datasets acquired from prostate tissues of NC, PCa, and PCa+MAT groups. Triplicate MALDI-MS acquisitions were performed per tissue-coated slide to ensure analytical reproducibility.

**FIGURE 2:**
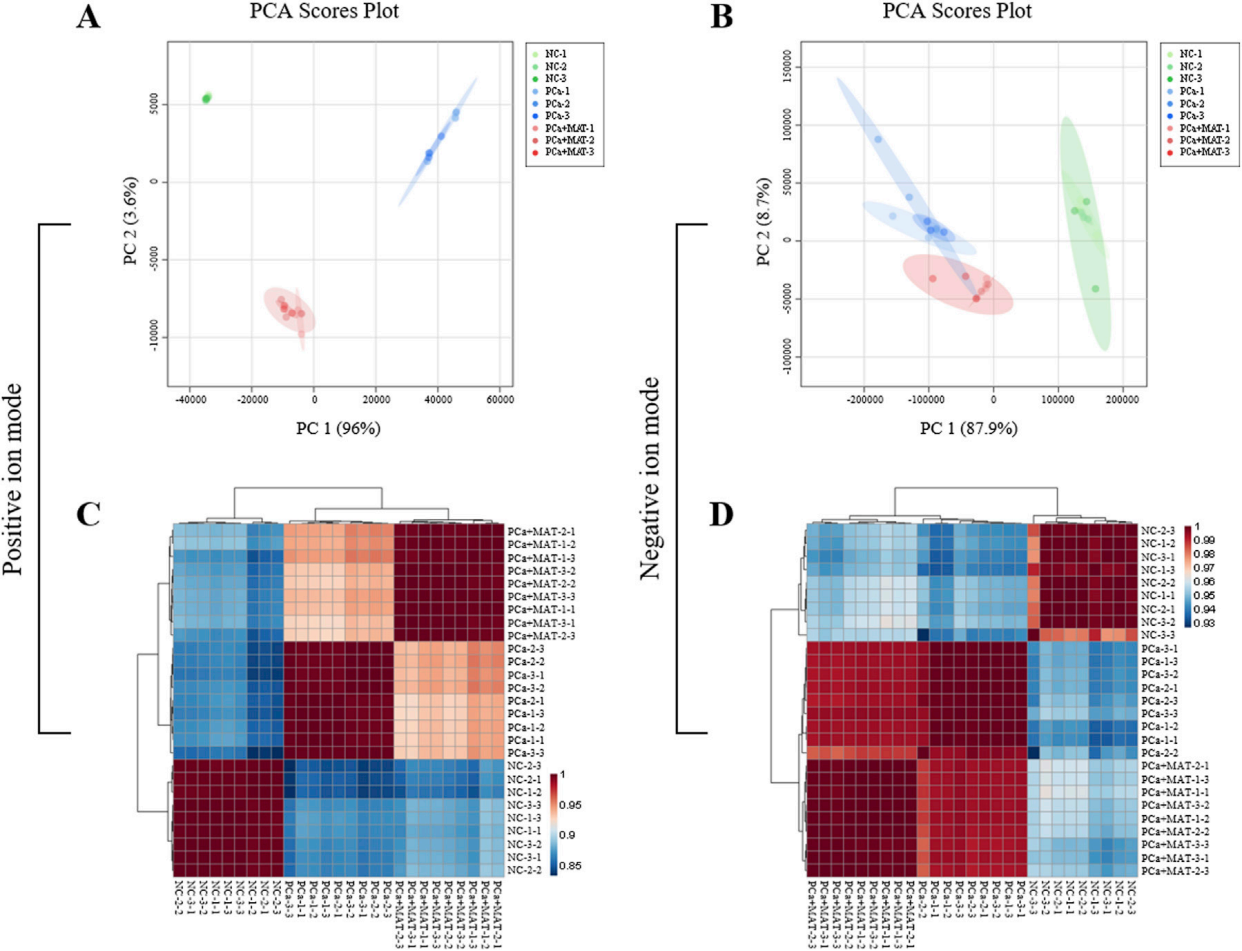
PCA and Pearson correlation-based clustering heatmap analysis to evaluate the LMW compounds changes in the prostate tissues from NC, PCa and PCa+MAT groups in the positive **(A,C)** and negative **(B,D)** ion modes.

In positive ion mode, PCA (Figure 2A) demonstrated clear separation along principal components PC1 (96% variance) and PC2 (3.6% variance), collectively explaining 99.6% of total metabolic variation. Corresponding negative ion mode analysis (Figure 2B) revealed similar clustering patterns with 96.6% cumulative variance (PC1:87.9%, PC2:8.7%). In positive ion mode analysis, Pearson correlation-based clustering heatmap (Figure 2C) revealed elevated intra-group correlations for NC, PCa, and PCa+MAT samples (PCC > 0.92), with comparable inter-group similarity between PCa and PCa+MAT groups (PCC > 0.92). Conversely, NC group demonstrated significantly lower correlations with both PCa and PCa+MAT groups (PCC < 0.92). This differential pattern persisted in negative ion mode (Figure 2D), though with higher correlation thresholds: intra-group and PCa-PCa+MAT inter-group PCC values exceeded 0.93, while NC-related inter-group correlations remained below this threshold. These findings collectively demonstrate significant alterations in endogenous LMW compound expression profiles across the experimental groups.

### 3.3 PLS-DA modeling for metabolic profiling of endogenous LMW compounds

**FIGURE 3:**
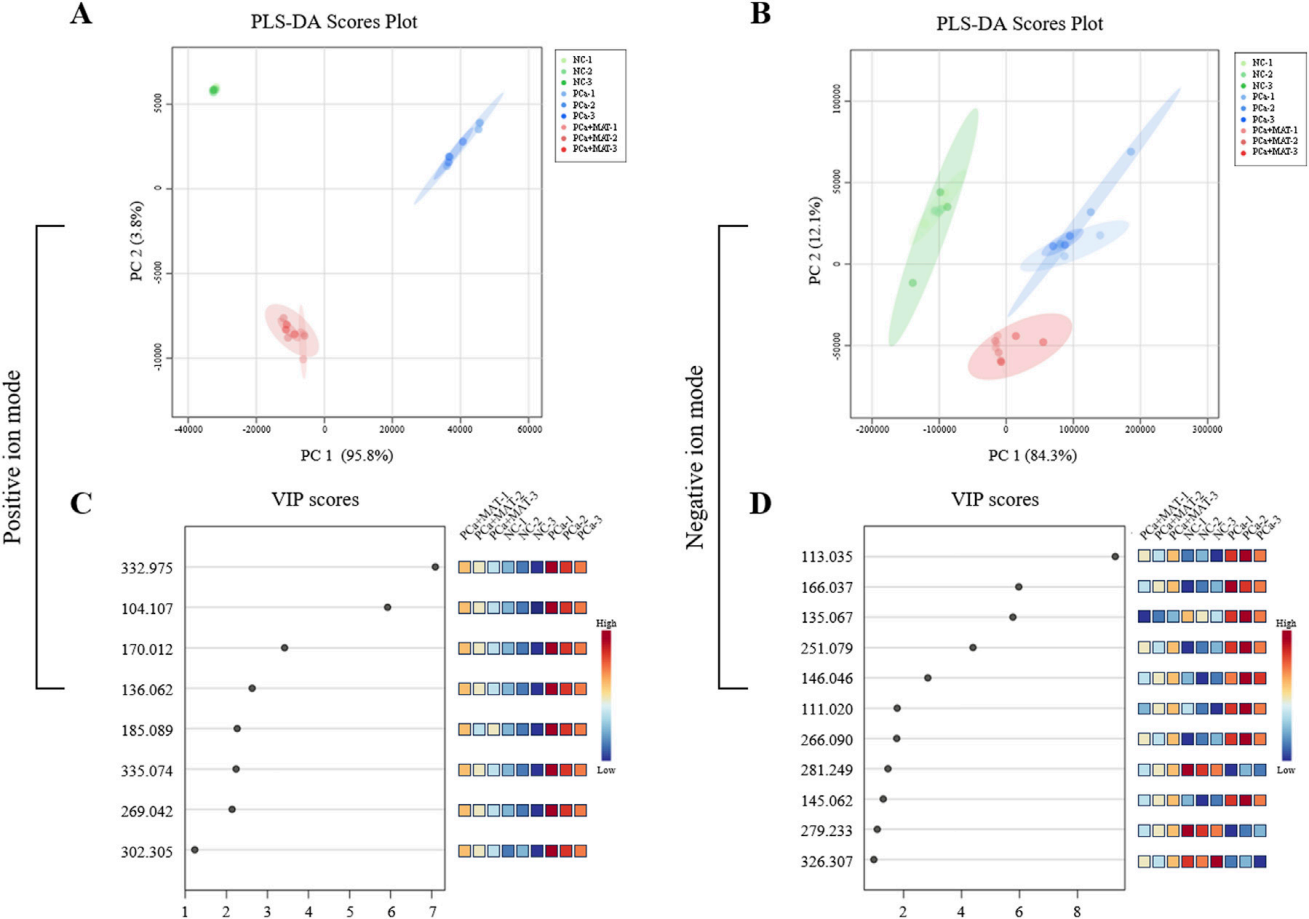
PLS-DA and VIP analysis to screen the significantly differential LMW compounds in the prostate tissues from NC, PCa and PCa+MAT groups in the positive **(A,C)** and negative **(B,D)** ion modes.

PLS-DA was implemented to characterize metabolic disparities between groups using *in situ* detected LMW compound profiles. The model demonstrated effective group separation in both ionization modes, with distinct clustering of NC, PCa, and PCa+MAT groups along PC1 and PC2 axes in three-dimensional score plots (Figures 3A,B).

Metabolites with variable importance in projection (VIP) scores >1.0 were identified as key discriminators (Table 1). Eight discriminant metabolites (VIP>1) were identified through positive ion mode analysis, with characteristic *m*/*z* values at 104.107, 136.062, 170.012, 185.089, 269.042, 302.305, 332.975, and 335.074 (Figure 3C). Complementarily, negative ion profiling detected eleven discriminatory ions spanning *m*/*z* 111.020–326.307, specifically *m*/*z* 111.020, *m*/*z* 113.035, *m*/*z* 135.067, *m*/*z* 145.062, *m*/*z* 146.046, *m*/*z* 166.037, *m*/*z* 251.079, *m*/*z* 266.090, *m*/*z* 279.233, *m*/*z* 281.249, and *m*/*z* 326.307 (Figure 3D).

The distinct LMW compound profiles identified through PLS-DA modeling suggest critical metabolic reprogramming events in prostate carcinogenesis. The 19 VIP-selected metabolites (8 in positive mode, 11 in negative mode) likely represent key nodes in matrine-mediated metabolic regulation of prostate cancer.

### 3.4 Structural identification and characteristics of differential expression of LMW compounds

The putative identification of differential metabolites was achieved through HMDB database matching (mass error <10 ppm). As shown in Table 1, metabolites identified in positive ion mode included choline (*m*/*z* 104.107), adenine (*m*/*z* 136.062), cysteic acid (*m*/*z* 170.012), 5-Hydroxylysine (*m*/*z* 185.089), glycerophosphoglycerol (GPG) (*m*/*z* 269.042), sphinganine (*m*/*z* 302.305), Ribose 1,5-bisphosphate, (Rib-1,5-P2) (*m*/*z* 332.975) and glycerophosphoinositol (GPI) (*m*/*z* 335.074), while negative ion mode detection confirmed uracil (*m*/*z* 111.020), dihydrouracil (*m*/*z* 113.035), tetrahydropteridine (BH_4_) (*m*/*z* 135.067), glutamine (*m*/*z* 145.062), glutamate (*m/z* 146.046), 8-Hydroxyguanine (8-OH-Gua) (*m*/*z* 166.037), deoxyinosine (*m*/*z* 251.079), adenosine (*m*/*z* 266.090), linoleic acid (*m*/*z* 279.233), oleic acid (*m*/*z* 281.249), N, N-Dimethylsphingosine (N,N-DMS) (*m*/*z* 326.307).

**FIGURE 4:**
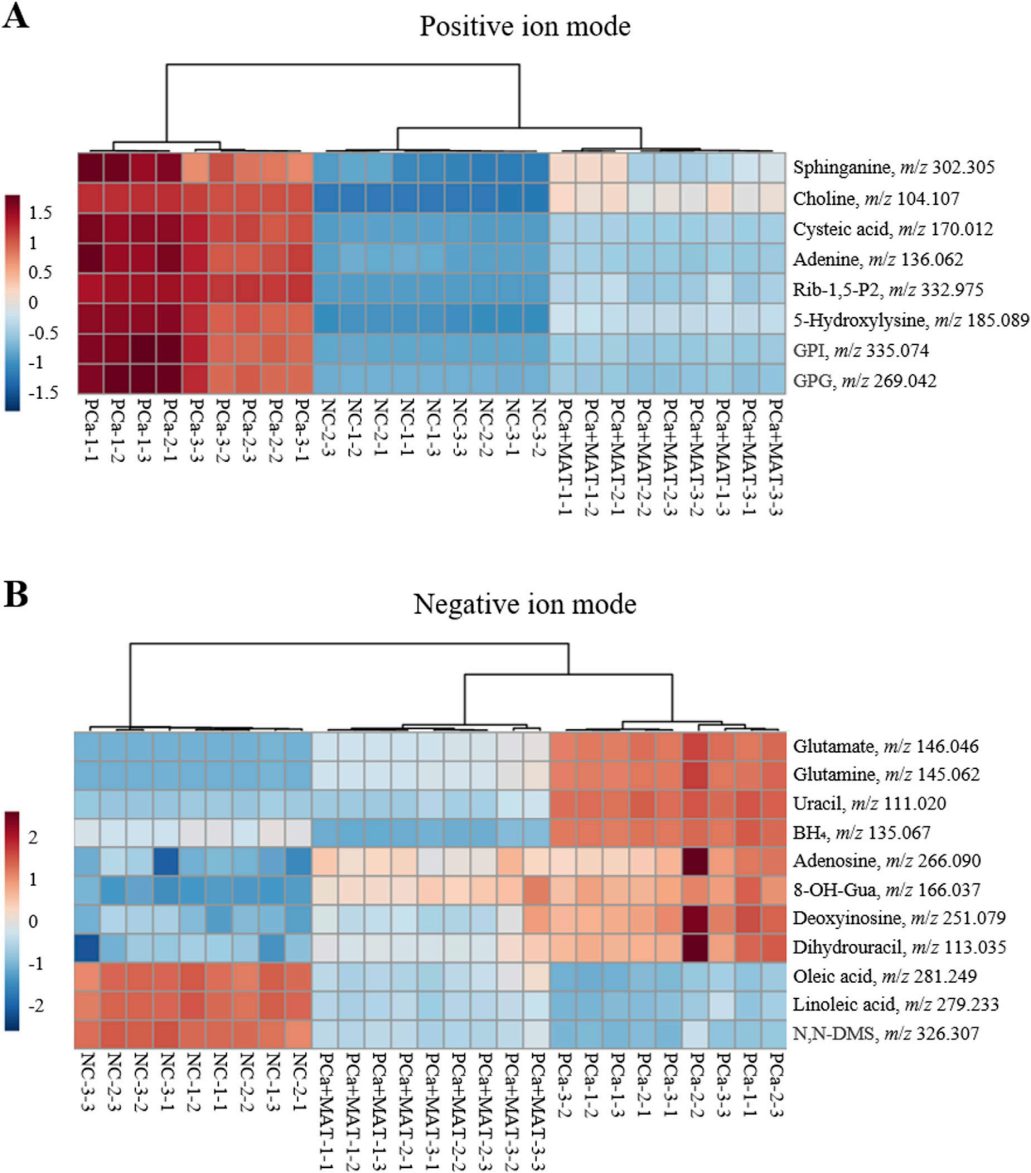
Hierarchical clustering heatmap analysis of the significantly changes of LMW compounds in the prostate tissues from NC, PCa and PCa+MAT groups in positive **(A)** and negative **(B)** ion modes.

**FIGURE 5:**
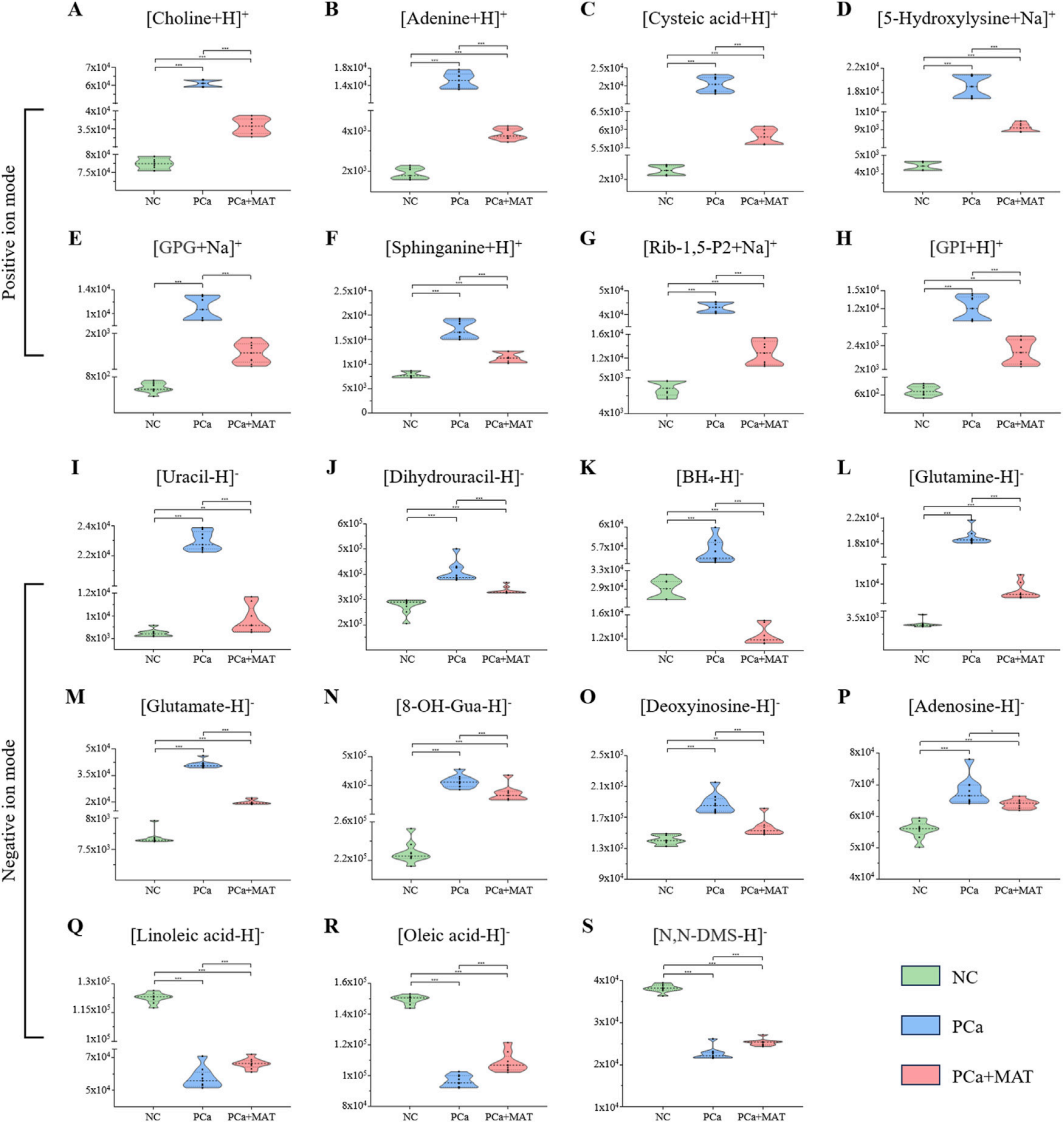
Violin plots showing the significantly changes of LMW compounds detected in the prostate tissues from NC, PCa and PCa+MAT groups by MALDI-TOF MS in positive **(A–H)** and negative **(I–S)** ion mode (“*”, 0.01 < *p* < 0.05; “**”, 0.001 < *p* < 0.01; “***”, *p* < 0.001).

The differential expression profiles of 19 LMW compounds across NC, PCa, and PCa+MAT groups, as visualized by hierarchical clustering heatmaps and violin plots, revealed distinct metabolic alterations associated with prostate cancer progression and the modulatory effects of matrine treatment. Hierarchical clustering heatmap analysis showed that linoleic acid, oleic acid and N,N-DMS were downregulated in the PCa group, while the remaining LMW compounds were upregulated (Figure 4). Violin plot more clearly showed that the expression levels of 15 LMW compounds were the lowest in the NC group, while they were significantly upregulated in the PCa group and with intermediate levels observed in the PCa+MAT group (Figures 5A–J,L–P). However, polyunsaturated fatty acids (e.g., linoleic acid, oleic acid) and N,N-DMS displayed an inverse pattern, with the highest levels in NC group, significant downregulation in PCa group, and partial restoration in PCa+MAT group (Figures 5Q–S). In addition, the results revealed a progressive depletion of BH_4_ across experimental groups, with the PCa+MAT cohort exhibiting significantly lower BH_4_ levels compared to both NC and PCa groups (PCa+MAT < NC < PCa; Figure 5K). This gradational depletion pattern suggests matrine intervention induces targeted modulation of BH_4_ biosynthesis or recycling pathways. These trend reflect carcinogenesis-driven alterations of pathway-specific metabolites, including lipid signaling mediators (e.g., choline, GPG, sphinganine, GPI, linoleic acid, oleic acid, N,N-DMS), amino acid network regulators (e.g., cysteic acid, 5-Hydroxylysine, glutamine-glutamate axis components), nucleotide biosynthesis (e.g., adenine, Rib-1,5-P2, uracil, dihydrouracil, deoxyinosine, adenosine), markers of oxidative damage (e.g., 8-OH-Gua) and cofactor of nitric oxide synthases (NOS) and aromatic amino acid hydroxylases (e.g., BH_4_), which are critical for sustaining proliferative signaling and metabolic reprogramming in prostate cancer cells. Notably, the partial recovery of these metabolites in the PCa+MAT group compared to the PCa group implies that matrine treatment partially reversed cancer-related metabolism.

### 3.5 Spatial distribution of LMW compounds in prostate tissues revealed by MALDI-MSI

MALDI-MSI was employed to spatially resolve the distribution of 19 LMW compounds in prostate tissues from NC, PCa, and PCa+MAT groups. Tissue sections were analyzed in both positive and negative ion modes using optimized matrices to enhance ionizability and spatial specificity. MALDI-MSI was performed at a spatial resolution of 75 μm, enabling precise mapping of metabolite distributions relative to tissue architecture.

**FIGURE 6:**
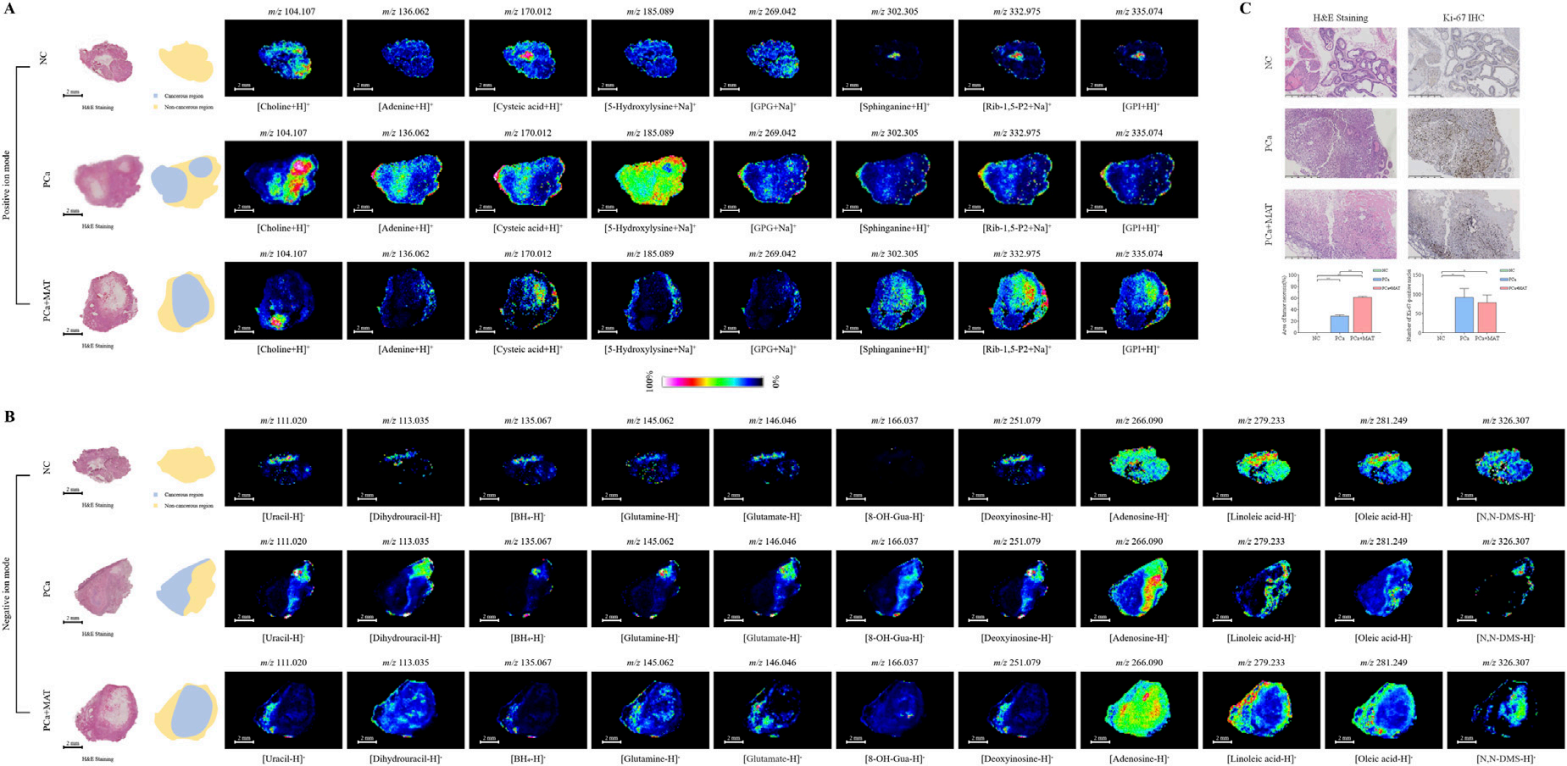
**(A)** Positive ion images of LMW compounds in prostate tissue sections from NC, PCa and PCa+MAT groups using 2-MBT as the matrix. Molecular images showing the distribution of different compounds ([M+H]^+^, [M+Na]^+^ and [M+K]^+^) across the intact prostate section surface. **(B)** Negative ion images of LMW compounds in prostate tissue sections from NC, PCa and PCa+MAT groups using MEK as the matrix. Molecular images showing the distribution of different compounds ([M-H]^−^) across the intact prostate section surface. H&E staining was performed after MSI. MSI was acquired at 75 μm spatial resolutions. Definition of prostate tissue on the section (blue: Cancerous regions; yellow: Non-cancerous regions). **(C)** Representative images of H&E staining and Ki-67 IHC staining in prostate tissue samples and their respective quantifications.

In positive ion mode (Figure 6A), molecular images acquired with 2-MBT as the matrix revealed distinct spatial patterns for LMW compounds detected as [M+H]^+^, [M+Na]^+^, and [M+K]^+^ adducts. These ions exhibited heterogeneous distribution across the intact prostate tissue surface, with pronounced localization differences between histologically annotated cancerous (blue) and non-cancerous (yellow) regions. In the PCa group, all eight LMW compounds showed elevated intensities in cancerous zones, correlating with their dysregulated expression profiles observed in bulk tissue analyses. Conversely, the PCa+MAT group displayed attenuated signal intensities for these LMW compounds in cancerous zones, suggesting a partial restoration of metabolic homeostasis following matrine treatment. In negative ion mode (Figure 6B), molecular imaging using MEK as the matrix resolved [M-H]^-^ adducts of metabolites. Linoleic acid, oleic acid, and N,N-DMS preferentially accumulate in NC tissues and tumor-specific downregulation is observed in PCa tissues, whereas the opposite is true for other LMW compounds. In PCa+MAT tissues, intermediate signal intensities were observed, indicating a regulatory effect of matrine on LMW compounds metabolism.

Post-imaging H&E staining confirmed the histological integrity of the analyzed sections and validated the annotated cancerous and non-cancerous regions. Histopathological evaluation of prostate tissues via H&E staining revealed distinct morphological differences across experimental groups (Figure 6C). In the NC group, tissue architecture remained intact with no evidence of tumorigenesis or necrosis. In contrast, PCa tissues exhibited characteristic tumor regions with disorganized glandular structures and focal areas of necrosis, consistent with aggressive tumor progression. Notably, PCa+MAT tissues displayed a significant increase in tumor necrosis area compared to the PCa group (*p* < 0.001), suggesting that matrine treatment may exacerbate tumor cell death or impair survival pathways in malignant cells. The absence of necrotic foci in NC tissues further validated the cancer-specificity of these observations. Ki-67 immunohistochemistry (IHC) staining was performed to assess cellular proliferation. Quantification of Ki-67-positive nuclei demonstrated a trend toward reduced proliferative activity in the PCa+MAT group compared to the PCa group. However, this difference did not reach statistical significance (*p* = 0.05), potentially due to sample heterogeneity or the partial nature of matrine’s anti-proliferative effects. In NC tissues, Ki-67 positivity was minimal, aligning with the quiescent state of normal prostate epithelium. The increased necrosis of tissues in PCa+MAT group, coupled with attenuated metabolic hyperactivity, implies that matrine may induce tumor cell death through multiple pathways. While Ki-67 results did not show significant suppression of proliferation, the partial reversal of cancer-related metabolism in the PCa+MAT group suggests that matrine’s therapeutic effects may prioritize metabolic reprogramming and necrosis induction over direct inhibition of proliferative signaling. This dichotomy underscores the complexity of matrine’s anti-tumor action, potentially involving multiple synergistic pathways.

## 4 Discussion

As fundamental architectural components of cellular membranes, lipids serve as critical mediators of intercellular communication, signaling transduction, and energy provision (Domingues et al., 2025; Loix et al., 2024). Lipid homeostasis maintains the dynamic equilibrium between biosynthesis and degradation, a process indispensable for cellular viability. Increasing evidence reveals that lipid metabolic reprogramming critically drives oncogenesis and tumor progression by dysregulating *de novo* lipogenesis, remodeling membrane architecture, and hijacking lipid-mediated signaling pathways, which collectively modulate hallmark malignancy features encompassing tumor microenvironment adaptation, metastatic dissemination, and therapeutic recalcitrance (Wu et al., 2024; Khan et al., 2024; Ward et al., 2025; Nicolini and Ferrari, 2024). In PCa, this metabolic rewiring is achieved through a multifaceted interplay of molecular regulators, collectively fostering a lipid-enriched microenvironment conducive to metastasis and therapeutic resistance (Wei et al., 2024; Ma et al., 2021; Deng et al., 2024). Our investigation identified significant disruptions in polyunsaturated fatty acid homeostasis within the PCa group, characterized by diminished levels of essential metabolic substrates including linoleic acid and oleic acid, findings that correspond to elevated lipid catabolism required for sustaining tumor bioenergetic demands. Critically, matrine intervention restored the depleted polyunsaturated fatty acid levels to some extent, demonstrating its capacity to partially reverse tumor-driven lipid metabolic reprogramming and reestablish lipid homeostasis. Furthermore, Sphingolipids are a class of lipids, including sphingolipids, ceramides and sphingosine-1-phosphate, which function as metabolic rheostats within the tumor microenvironment, orchestrating critical oncogenic processes through their dynamic interconversion (Gregg et al., 2024). This sphingolipid signaling axis modulates malignant cell survival/apoptosis balance, neovascularization patterns, migratory plasticity, and therapeutic responsiveness, thereby serving as a key node integrating metabolic reprogramming with tumor progression (Zhou et al., 2024; Mellor et al., 2024). In this study, the concurrent depletion of the sphingosine derivative N,N-DMS and accumulation of sphinganine observed in the PCa group reflects dysregulation at two critical nodes of sphingolipid metabolism, wherein elevated sphinganine levels indicate augmented sphingolipid biosynthesis while diminished N,N-DMS suggests impaired metabolic flux through methylation-dependent conversion pathways. This dual dysregulation may mechanistically activates the sphingosine-1-Phosphate (S1P) signaling. Given that S1P is a well-established activator of the PI3K/AKT pathway via its cognate receptors (Rostami et al., 2019; Dilber et al., 2024), we propose that this specific pattern of metabolite changes collectively fosters a microenvironment conducive to the activation of the PI3K/AKT pathway, thereby promoting cell survival and tumor progression. Interestingly, matrine intervention showed partial metabolic recovery, suggesting that its anti-tumor effects may be mediated, at least in part, through the normalization of sphingolipid metabolism. Collectively, these metabolic findings and our prior transcriptomic data (Li et al., 2018) support a model in which matrine exerts its anti-tumor effect by normalizing sphingolipid metabolism, thus suppressing the oncogenic S1P-PI3K/AKT signaling axis. The direct validation of this proposed mechanism constitutes a key objective of our ongoing research. Phospholipids play pivotal roles in maintaining membrane structural stability, facilitating signal transduction, and regulating energy metabolism (Yi et al., 2021). This study reveals aberrant glycerophospholipid metabolism in PCa, characterized by elevated expression levels of choline, GPG, and GPI—key intermediates in the phosphatidylcholine biosynthetic pathway. Notably, matrine treatment in the PCa+MAT group elicited a marked attenuation of these metabolic perturbations, as evidenced by substantial reductions in choline, GPG, and GPI levels compared to the PCa group. The observed metabolic reversion underscores matrine’s potential to counteract cancer-associated phospholipid dysregulation through targeted modulation of membrane biosynthesis pathways.

Emerging evidence reveals that amino acids not only fuel malignant progression through provisioning biosynthetic precursors for accelerated protein synthesis and dysregulated cell proliferation, but also mechanistically drive tumor invasion programs, metastatic niche formation, and immunosuppressive microenvironment remodeling via metabolic reprogramming (Butler et al., 2021; Yao et al., 2025). Of particular note, glutamine serves as the predominant anaplerotic substrate in cancer metabolism, functioning not merely as a bioenergetic fuel but critically enabling metabolic plasticity through its catabolic conversion into α-ketoglutarate–a pivotal tricarboxylic acid (TCA) cycle intermediate that bridges nitrogen balance with epigenetic regulation and redox homeostasis (Thiruvalluvan et al., 2022; Zhao et al., 2023). There are studies have shown that hyperactive glutaminolysis in PCa fuels the TCA cycle, potentiates oxidative phosphorylation-driven energy metabolism and fulfills heightened biosynthetic demands, which are closely strongly correlated with advanced-stage progression, therapeutic refractoriness and metastatic dissemination (Xu et al., 2021; Moon et al., 2024; Bhowmick et al., 2023). These pathophysiological features underscore the therapeutic potential of targeting glutaminolytic pathways. Intriguingly, our metabolomic analyses revealed marked depletion of both glutamine and its metabolic derivative glutamate in the PCa+MAT group relative to the PCa group, providing evidence that matrine inhibits glutamine metabolism in prostate cancer cells by specifically targeting glutaminolysis pathways and ultimately inhibited prostate cancer progression.

Nucleotides serve as essential components for cellular viability, with purine and pyrimidine biosynthesis pathways exhibiting multi-tiered regulatory mechanisms to maintain metabolic homeostasis (Tran et al., 2024; Sahu et al., 2024). Notably, rapidly proliferating cells demonstrate increased reliance on nucleotide anabolism, particularly through *de novo* synthesis pathways. This metabolic adaptation is characteristically observed in neoplastic cells, where elevated requirements for nucleic acid precursors drive enhanced utilization of nucleotide biosynthesis machinery to support uncontrolled proliferation (Lin et al., 2024). Our metabolomic profiling revealed significant upregulation of key nucleotide intermediates in PCa tissues, including adenine, uracil, dihydrouracil, deoxyinosine, and adenosine. This metabolic signature aligns with the enhanced nucleotide anabolism typically observed in neoplastic proliferation. Specifically, the accumulation of purine derivatives and pyrimidine-related metabolites suggests coordinated activation of both salvage and *de novo* synthesis pathways to meet the heightened demand for DNA/RNA precursors. Notably, matrine treatment effectively attenuated these metabolic perturbations, as evidenced by the systemic reduction of these nucleotide intermediates in PCa+MAT group. This therapeutic response supports the hypothesis that matrine may target the regulatory nodes of nucleotide homeostasis—potentially through modulating purine/pyrimidine biosynthesis signaling pathways—thereby restricting the metabolic plasticity essential for malignant progression.

Elevated oxidative stress emerged as a critical pathobiological determinant in carcinogenesis, manifested through distinct metabolic perturbations (Vahidi et al., 2024; Lior et al., 2024). In this study, the observed elevation of cysteic acid, 8-OH-Gua and Rib-1,5-P2 in PCa group provides mechanistic insights into oxidative pathophysiology in PCa progression. Cysteic acid represents the terminal oxidative derivative in the cysteine oxidation cascade, resulting from further oxidation from cysteine sulfinic acid. Cysteic acid is considered an irreversible post-translational modification, which serves as a molecular signature of oxidative stress that has resulted in oxidative damage to proteins (Bhatt and Zondlo, 2023). 8-OH-Gua upregulation directly reflects guanine oxidation, indicating that the DNA repair capacity of neoplastic cells is impaired because of ROS attack on DNA (Jelic et al., 2021). Rib-1, 5-P2 is an intermediate metabolite of the pentose phosphate pathway (PPP) (TeSlaa et al., 2023), and its upregulation reflects the enhanced NADPH synthesis by tumor cells through PPP to resist reactive oxygen species (ROS), which may lead to the accumulation of intermediates due to metabolic imbalance, suggesting the metabolic reprogramming state of the tumor. Notably, matrine’s concurrent attenuation of these oxidative-metabolic biomarkers effectively reverses pathological alterations, indicating a multimodal therapeutic mechanism that targets critical regulatory nodes at the metabolic and oxidative systems.

In addition, the observed BH_4_ depletion in PCa+MAT group may reflect dual regulatory effects within the tumor microenvironment. As an essential cofactor for NOS and aromatic amino acid hydroxylases, BH_4_ reduction could mechanistically (a) attenuate NOS-derived nitric oxide overproduction associated with tumor angiogenesis, and (b) preserve neurotransmitter homeostasis by maintaining tyrosine/tryptophan hydroxylase activity (Gonçalves et al., 2021). Our data further propose that matrine’s antitumor action may involve selective restriction of pro-tumorigenic BH_4_ pools while sustaining neuroendocrine functions.

Collectively, this study demonstrates that prostate carcinogenesis is underpinned by dynamic dysregulation of interconnected metabolic axes-particularly involving lipid remodeling, amino acid/nucleotide flux and oxidative stress-which cooperatively fuel malignant progression through tumor microenvironment remodeling. The spatially resolved enrichment of LMW compounds reflects an inherent metabolic compartmentalization during prostate carcinogenesis. This compartmentalization likely enables tumor cells to allocate resources for progression (e.g., biological synthesis) while suppressing anti-tumor signaling. Therapeutically, matrine modulates the spatial distribution of these metabolites to perturb the tumor’s adaptive metabolic zonation-a mechanistically coherent phenomenon that may underlie its anti-neoplastic efficacy. Notably, matrine exerts preferential regulatory effects on malignancy-associated lipid remodeling networks, specifically manifesting in the metabolic normalization of polyunsaturated fatty acid metabolism, sphingolipid biosynthesis, and glycerophospholipid homeostasis - emerging as hallmark therapeutic responses. These coordinated metabolic corrections collectively validate matrine’s capacity to reprogram oncogenic metabolism through targeted pathway intervention. Consequently, systematic interrogation on the role of LMW compounds in metabolite-pathways such as lipid metabolic remodeling, amino acid/nucleotide synthesis and oxidative stress regulation will be critical for deciphering the spatiotemporal dynamics underlying matrine’s multidimensional antitumor efficacy.

Despite these insights, several limitations warrant consideration in this study. Firstly, the study focused exclusively on LMW compounds, leaving the roles of high-molecular-weight (HMW) metabolites (e.g., HMW proteins and lipids), unexplored. Integrating proteomic or lipidomics analyses will provide a more holistic understanding of matrine’s therapeutic mechanisms. Additionally, while our findings highlight matrine’s pronounced modulation of lipid metabolism, the precise molecular targets and regulatory pathways underlying this effect remain to be elucidated. Future studies should prioritize lipid-centric investigations, including lipidomic profiling and functional validation of key enzymes (e.g., sphingolipid synthases or fatty acid desaturases). Moreover, the xenograft model employed here may not fully recapitulate the heterogeneity of human prostate cancer, necessitating validation in patient-derived models or clinical cohorts. Addressing these gaps will refine our understanding of matrine’s anticancer potential and accelerate its translation into targeted therapies. Nevertheless, this study establishes MALDI-MSI as a powerful platform for pharmacometabolomic evaluation, while providing mechanistic insights into matrine’s anticancer action and validate metabolomic approaches for natural product evaluation.

## 5 Conclusion

In this study, we used MALDI-MSI-based *in situ* detection to investigate the response of prostate cancer to matrine treatment. Our results reveal that the metabolic landscape of prostate cancer is dynamically reshaped during disease progression, with matrine exerting modulatory effects on pathways central to lipid metabolic remodeling, amino acid/nucleotide synthesis and oxidative stress regulation. The intermediate metabolite levels in PCa+MAT group further highlight the potential of matrine to recalibrate oncogenic metabolic networks without fully restoring baseline, possibly due to incomplete pathway inhibition or compensatory mechanisms. Further investigation into the crosstalk between these metabolites and signaling cascades may elucidate the mechanistic basis of matrine’s therapeutic action.

## Data Availability

The original contributions presented in the study are included in the article/supplementary material, further inquiries can be directed to the corresponding authors.
